# Impact of circumferential resection margin on survival in ampullary cancer: retrospective analysis

**DOI:** 10.1093/bjsopen/zrad120

**Published:** 2023-12-29

**Authors:** Anna Nießen, Martin Loos, Katja Neumüller, Manuel Feißt, Ulla Klaiber, Amila Cizmic, Mohammed Al-Saeedi, Susanne Roth, Martin Schneider, Markus W Büchler, Thilo Hackert

**Affiliations:** Department of General, Visceral and Transplantation Surgery, Heidelberg University Hospital, Heidelberg, Germany; Department of General, Visceral and Thoracic Surgery, University Hospital Hamburg-Eppendorf, Hamburg, Germany; Department of General, Visceral and Transplantation Surgery, Heidelberg University Hospital, Heidelberg, Germany; Department of General, Visceral and Transplantation Surgery, Heidelberg University Hospital, Heidelberg, Germany; Institute of Medical Biometry, Heidelberg University Hospital, Heidelberg, Germany; Department of General Surgery, Division of Visceral Surgery, Medical University of Vienna, Vienna, Austria; Department of General, Visceral and Transplantation Surgery, Heidelberg University Hospital, Heidelberg, Germany; Department of General, Visceral and Thoracic Surgery, University Hospital Hamburg-Eppendorf, Hamburg, Germany; Department of General, Visceral and Transplantation Surgery, Heidelberg University Hospital, Heidelberg, Germany; Department of General, Visceral and Transplantation Surgery, Heidelberg University Hospital, Heidelberg, Germany; Department of General, Visceral and Transplantation Surgery, Heidelberg University Hospital, Heidelberg, Germany; Department of General, Visceral and Transplantation Surgery, Heidelberg University Hospital, Heidelberg, Germany; Botton-Champalimaud Pancreatic Cancer Center, Champalimaud Foundation, Lisbon, Portugal; Department of General, Visceral and Transplantation Surgery, Heidelberg University Hospital, Heidelberg, Germany; Department of General, Visceral and Thoracic Surgery, University Hospital Hamburg-Eppendorf, Hamburg, Germany

## Abstract

**Background:**

Ampullary carcinoma is a clinically variable entity. This study aimed to evaluate prognostic factors for the outcome of resected ampullary carcinoma patients with particular intent to analyse the influence of surgical radicality.

**Methods:**

Patients undergoing resection between 2002 and 2017 were analysed. Clinicopathological parameters, perioperative outcome and survival were examined. Risk factor analysis for postresection survival was performed. Resection margin status was evaluated according to the revised classification for pancreatic adenocarcinoma.

**Results:**

A total of 234 patients were identified, 97.9 per cent (*n* = 229) underwent formal resection, while 2.1 per cent (*n* = 5) underwent ampullary resection. Histological subtypes were 46.6 per cent (*n* = 109) pancreatobiliary, 34.2 per cent (*n* = 80) intestinal, 11.5 per cent (*n* = 27) mixed, and 7.7 per cent (*n* = 18) undetermined. In the pancreatobiliary group, tumours were more advanced with more vascular resections, pT4 stage, G3 differentiation and pN+ status. Five-year overall survival was significantly different for pancreatobiliary compared to intestinal (51.7 per cent *versus* 72.8 per cent, *P* = 0.0087). In univariable analysis, age, pT4 stage, pN+, pancreatobiliary subtype and positive resection margin were significantly associated with worse overall survival. Long-term outcome was significantly better after true R0 resection (circumferential resection margin–, tumour clearance >1 mm) compared with circumferential resection margin+ (<1 mm) and R1 resections (5-year overall survival: 69.6 per cent, median overall survival 191 months *versus* 42.4 per cent and 53 months; *P* = 0.0017).

**Conclusion:**

Postresection survival of ampullary carcinoma patients is determined by histological subtype and surgical radicality. Intestinal differentiation is associated with less advanced tumour stages and better differentiation, which is reflected in a significantly better overall survival compared to pancreatobiliary differentiation. Despite this, true R0-resection is a prognostic key determinant in both entities, achieving 5-year survival in two-thirds of patients.

## Teaser

Ampullary carcinoma is a rare tumour entity with different histological subtypes. Identification of risk factors influencing postresection survival is essential for treatment strategies. True R0-resection is a key determinant associated with excellent postresection survival.

## Introduction

Ampullary carcinoma (AC) is a rare and heterogeneous clinical entity accounting for 0.2 per cent of all gastrointestinal tumours^1^. Due to its location at the transitional zone between the small bowel, pancreas and bile duct, the different epithelial linings in case of neoplastic transformation can give rise to a number of different histopathological subtypes^2^. At the moment, AC is classified according to the 8th edition of the TNM classification of malignant tumours regardless of its histopathological differentiation^3^.

AC is thought to have a better prognosis than pancreatic ductal adenocarcinoma (PDAC) or distal bile duct carcinoma, partly due to its localization at the Papilla Vateri, which leads to characteristic symptoms such as jaundice and pain. Five-year overall (OS) and disease-free survival rates (DFS) of resected patients are reported around 50 per cent^4^. Differences with regards to prognosis and treatment strategies have also been attributed to the different histopathological subtypes. While some studies reported patients with a pancreatobiliary (PB) subtype to have a significantly poorer 5-year OS and DFS than those with intestinal (INT) differentiation^5–7^ and PB subtype to be an independent predictor for poor survival^6^, some others have shown that the histological subtype is not an independent factor when accounting for stage^8,9^.

Data on prognostic factors and outcome of this rare disease is scarce. The most important predictors of survival identified are positive lymph node status (pN+)^1,10,11^ and lymph node ratio (LNR)^10–12^. The role of adjuvant therapy is still a matter of debate and current literature is diverse^7,13–18^. Data on the role and impact of the resection margin status (R status) is limited with some reports showing association with worse survival^14,19^, but none evaluating the circumferential resection margin (CRM).

The aim of this study was to analyse the long-term outcome of resected AC focusing on differences between histological subtypes and risk factors for survival.

## Methods

### Patient identification

This study was approved by the ethics committee of the Medical Faculty of the University of Heidelberg (no. S-140/2019). All patients undergoing resection of AC between 1 January 2002 and 31 December 2017 were retrospectively identified from a prospectively maintained database at the Department of General, Visceral and Transplantation Surgery, University Hospital of Heidelberg, Germany.

### Clinical data and surgical approach

Patient data were extracted from the database and the hospital’s digital patient information system. Parameters collected included: age, sex, type of operation, duration of operation, duration of hospital stay and ICU stay and neo-/adjuvant chemotherapy, postoperative pancreatic fistula (POPF) according to the International Study Group of Pancreatic Surgery (ISGPS) classification^20,21^, delayed gastric emptying (DGE)^22^, wound infection, sepsis^23^, reoperation rate, postoperative haemorrhage and 90-day mortality rate, histological subtype, R status, tumour size, distant metastases, and TNM stage and grading according to the 8th TNM classification^3^. R status was evaluated according to the revised classification for PDAC based on the Leeds protocol for histopathological workup^24^. Radicality was stratified as R0 CRM– (tumour clearance >1 mm), R0 CRM+ (tumour clearance <1 mm) and R1 (direct tumour infiltration of the resection margin). Patients missing information on the R status or the CRM status were re-evaluated by a senior pathologist.

### Follow-up

Follow-up data were retrieved from the outpatient care unit, the external oncological follow-up appointments and/or the residents’ registration offices. Telephone interviews with general practitioners and/or patients were also performed. Patients were followed until their latest oncological follow up visit or until death. Follow-up data included recurrences, application of adjuvant treatment and in case of death the cause of death.

### Statistical analysis

Statistical analysis was performed using the statistic software language R Version >4.2.0 in RStudio^25^. Quantitative parameters are presented as median with interquartile range (i.q.r.). Qualitative parameters are presented by absolute and relative frequencies. For subgroup comparisons, F-tests and *t* tests were used for quantitative and chi-square tests for qualitative parameters. OS, defined as the time from resection to either death from any cause or until the last follow-up, was analysed using the Kaplan–Meier method. Patients alive at last follow-up were censored. Patients with a follow-up of less than 90 days and those with distant metastases were excluded from the survival analysis. The log rank test was used for comparison of survival curves between subgroups. To identify risk factors correlated with OS, uni- and multivariable survival analysis was performed using the proportional hazard regression (Cox model). The hazard ratios (HR) and their 95 per cent confidence intervals (c.i.) are presented. Two-sided *P* values of <0.05 were considered statistically significant.

## Results

### Patient characteristics and perioperative data

A total of 234 patients undergoing resection for AC were identified. Clinicopathological and operative characteristics are presented in *Table 1*. Median age at operation was 67 years (i.q.r. 59–74). The male:female ratio was almost 2:1 (148 male *versus* 86 female patients). In 9 patients (3.8 per cent) neoadjuvant treatment was administrated. Some 82 (35.0 per cent) patients underwent preoperative stent placement. Preoperative tumour marker levels for CA 19-9 and CEA were 22 U/ml (i.q.r. 12–55) and 1.4 µg/l (i.q.r. 0.8–2.2) respectively. Overall, 229 (97.9 per cent) patients underwent formal oncological resection. The majority of patients (*n* = 215; 91.9 per cent) underwent partial pancreatoduodenectomy (PD). Fourteen patients (6.0 per cent) underwent total pancreatectomy (TP) and 5 (2.1 per cent) ampullary resection (AR). Some 16 patients (6.8 per cent) underwent vascular resection.

**Table 1:** Clinicopathological and perioperative characteristics of resected ampullary carcinoma patients (2002–2017)

Parameter	Total (*n* = 234)
**Age (years),** median (i.q.r.)*	67 (59–74)
**Neoadjuvant treatment** (no:yes)	225 (96.2):9 (3.8)
**Preoperative stent implantation** (no:yes) (2 missing values)	150 (64.1):82 (35.1)
**Type of surgery**	
PD	215 (91.9)
TP	14 (6.0)
AR	5 (2.1)
**Vascular resection** (no:yes)	218 (93.2):16 (6.8)
**T status (TNM 8th), n (%)** (4 missing values)	
T1	120 (8.5)
T2	90 (38.5)
T3	39 (16.7)
T4	80 (34.2)
Tis	5 (2.1)
**Nodal status**	
pN0	103 (44.0)
pN+	126 (53.9)
pNx	5 (2.1)
**ELN,** median (i.q.r.)	23 (16–30)
**PLN (% of ELN),** median (i.q.r.)	4.5 (0–21)
**Grading (WHO 2017),** (9 missing values)	
G1	14 (6.2)
G2	140 (62.2)
G3	71 (31.6)
**Distant metastasis** (M0:M1)	222 (94.9):12 (5.1)
**Resection margin status**	
R0 (CRM−)	175 (74.8)
R0 (CRM+)	23 (9.8)
R1	33 (14.1)
Rx	3 (1.3)
**Length of ICU stay (days),** median (i.q.r.)	2 (0–5)
**POPF**	47 (20.1)
Type B	24 (51.0)
Type C	23 (48.9)
**DGE** (no:yes) (7 missing values)	180 (76.9):47 (20.1)
**Wound infection** (no:yes) (5 missing values)	190 (83.0):39 (17.0)
**Sepsis** (no:yes)	210 (89.7):24 (10.3)
**Reoperation** (no:yes)	190 (81.2):44 (18.8)
**Postoperative haemorrhage** (no:yes)	212 (90.6):22 (9.4)
**90-day mortality rate**	13 (5.6)

Values are n (%) unless otherwise indicated. i.q.r., interquartile range; PD, partial pancreatoduodenectomy; TP, total pancreatectomy; AR, ampullary resection; ELN, examined lymph nodes; PLN, positive lymph nodes; CRM, circumferential resection margin; *at the time of operation; POPF, postoperative pancreatic fistula; DGE, delayed gastric emptying.

Some 126 (53.9 per cent) had nodal positive disease and 103 (44.0 per cent) were nodal negative with a median number of 23 examined lymph nodes (ELN; i.q.r. 16–30). Twelve patients (5.1 per cent) had distant metastases at the time of presentation. Four patients had a single liver metastasis that was excised, six patients had non-regional lymph node (LN) metastases that were resected. In one patient localized peritoneal carcinomatosis was discovered intraoperatively and excised. One patient underwent emergency resection of the pancreatic head (PD) due to diffuse bleeding from the duodenum which was not treatable endoscopically. In this patient, tumour diagnosis was made after surgery and diffuse liver metastases were seen upon staging. In 198 patients microscopically, clear resection margin (R0) was reached, while 33 (14.1 per cent) were R1. There were 175 of 234 patients with R0 CRM– (74.8 per cent) and 23 (9.8 per cent) with R0 CRM+ . Information on adjuvant treatment was available for 159 patients, of whom 72 (45.3 per cent) received adjuvant therapy.

In terms of postoperative complication rates (*Table 1*), POPF occurred in 47 patients (20.1 per cent), of whom 24 (51 per cent) developed POPF type B and 23 (48.9 per cent) POPF type C. In 44 patients (18.8 per cent), reoperation was necessary. Some 22 patients (9.4 per cent) developed postoperative haemorrhage. The 90-day mortality rate was 5.6 per cent (*n* = 13).

### Tumour stages and histological subtypes

In 80, 109 and 27 patients an INT, PB and mixed subtype was identified respectively (*Table 2*). In 18 patients, the histological subtype was either not available or different from the above mentioned categories, for example signet cell. These patients were excluded from this analysis. There was no significant difference in median age (*P* = 0.836), gender (*P* = 0.146), duration of hospital stay (*P* = 0.626), operation (*P* = 0.937) and ICU stay (*P* = 0.670) between the three subgroups. Median preoperative CA 19-9 was lower in the INT than in the PB subtype (16 U/ml, i.q.r. 11–38 *versus* 31 U/ml, i.q.r. 12–99) (*P* = 0.430). There was no difference in preoperative median CEA. Preoperative stent placement was performed in 21 (26.6 per cent) patients with INT subtype compared to 12 (44.4 per cent) in the mixed group and 42 (38.5 per cent) in the PB group (*P* = 0.178). Neoadjuvant treatment was performed in 2 (2.5 per cent) patients in the INT group, 4 (3.7 per cent) in the PB and 1 (3.7 per cent) in the mixed group (*P* = 0.895). There was no significant difference in adjuvant treatment.

**Table 2:** Clinicopathological characteristics and comparison between histological subtypes

Characteristic	Intestinal type (*n* = 80)	Pancreatobiliary type (*n* = 109)	Mixed type (*n* = 27)	*P*
**Age (years),** median (i.q.r.)	65 (59–73)	68 (58–75)	70 (59–74)	0.836
**Sex**				0.146
Male	45 (56.2):	76 (69.7):	16 (59.2):		
Female	35 (43.8)	33 (30.3)	11 (40.7)		
**Preoperative CEA (U/ml),** median (i.q.r.)	1.4 (0.7–2.2)	1.5 (0.9–2.2)	1.2 (0.45–2.2)	0.851
**Neoadjuvant treatment**	2 (2.5)	4 (3.7)	1 (3.7)	0.895
**Preoperative stent placement** (2 missing values)	21 (26.6)	42 (38.5)	12 (44.4)	0.178
**Type of surgery**				0.202
PD	78 (97.5)	97 (89.0)	24 (88.9)	
TP	1 (1.2)	10 (9.2)	2 (7.4)	
AR	1 (1.2)	2 (1.8)	1 (3.7)	
**Vascular resection**	3 (3.8)	9 (8.3)	4 (14.8)	0.147
**Length of operation** (min), median (i.q.r.)	300 (250–360)	300 (250–360)	300 (240–330)	0.937
**T status** (4 missing values)				0.042*
pT1	10 (12.5)	3 (2.8)	1 (3.7)	
pT2	32 (40.0)	36 (33.0)	15 (55.6)	
pT3	15 (18.8)	20 (18.3)	2 (7.4)	
pT4	22 (27.5)	49 (45.0)	9 (33.3)	
Tis	1 (1.2)	1 (0.9)	0 (0.0)	
**Nodal status** (2 missing values)				0.004*
pN0	45 (56.3)	35 (32.1)	12 (44.4)	
pN+	34 (42.5)	72 (66.1)	14 (51.9)	
pNx	1 (1.3)	2 (1.8)	1 (3.7)	
**ELN,** median (i.q.r.)	22 (18–28)	24 (18–31)	20 (16–30)	0.795
**PLN (% of ELN),** median (i.q.r.)	0 (0–17)	8 (0–26)	5 (0–25)	0.016*
**Distant metastasis**				0.054
M0	78 (97.5)	103 (94.5)	23 (85.2)	
M1	2 (2.5)	6 (5.5)	4 (14.8)	
**Tumour grade** (9 missing values)				0.002*
G1	8 (10.4)	2 (1.9)	0 (0.0)	
G2	54 (70.1)	60 (56.1)	19 (73.1)	
G3	15 (19.5)	45 (42.1)	7 (26.9)	
**Tumour size (cm),** median (i.q.r.)	2.6 (1.8–3.5)	2 (1.5–2.5)	2.2 (2–2.5)	0.007*
**Resection margin status**				0.064
R0 CRM–	67 (83.8)	75 (68.8)	17 (63.0)	
R0 CRM+	4 (5.0)	15 (13.8)	3 (11.1)	
R1	7 (8.8)	19 (17.4)	6 (22.2)	
Rx	2 (2.5)	0 (0)	1 (3.7)	
**Length of ICU stay (days),** median (i.q.r.)	2 (0–8)	2 (0–4)	2 (0–5)	0.670
**90-day mortality rate**	2 (2.5)	11 (10.1)	0 (0.0)	0.035*
**POPF**	17 (21.2)	19 (17.4)	5 (18.5)	0.802
**DGE** (7 missing values)	14 (17.5)	26 (23.9)	5 (18.5)	0.531
**Wound infection** (5 missing values)	10 (12.7)	20 (19.0)	5 (18.5)	0.493
**Sepsis**	6 (7.5)	16 (14.7)	1 (3.7)	0.131
**Reoperation**	11 (13.8)	23 (21.1)	5 (18.5)	0.430
**Postoperative haemorrhage**	7 (8.7)	12 (11.0)	2 (7.4)	0.796
**Adjuvant therapy,** *n* ^†^	22	37	8	0.275

Values are n (%) unless otherwise indicated. *Statistically significant; ^†^of 144 patients with available information on adjuvant therapy and these subtypes; i.q.r., interquartile range; CEA, carcinoembryonic antigen; PD, partial pancreatoduodenectomy; TP, total pancreatectomy; AR, ampullary resection; ELN, examined lymph nodes; PLN, positive lymph nodes; CRM, circumferential resection margin; n.a., not available or other than intestinal or pancreatobiliary subtype; POPF, postoperative pancreatic fistula.

Patients in the PB group had more advanced tumours with more pT4 stages (n = 49 (45 per cent) versus 22 (27.5 per cent); *P* = 0.042), G3 differentiation (*n* = 45 (42.1 per cent) versus 15 (19.5 per cent); *P* = 0.002) and pN+ patients (72 (66.1 per cent) versus 34 (42.5 per cent); *P* = 0.004) with similar rates of ELN (22, i.q.r. 18–28 versus 24, i.q.r. 18–31) compared to the INT group. Interestingly, there was a significant difference in median tumour size: 2.6 cm (i.q.r. 1.8–3.5) in the INT compared with 2 cm (i.q.r. 1.5 −2.5) in the PB and 2.2 cm (i.q.r. 2–2.5) in the mixed group (*P* = 0.007).

R0 CRM– resection was reached in 67 (83.8 per cent) of INT compared with 75 (68.8 per cent) PB and 17 (63.0 per cent) mixed type patients. R0 CRM+ was reached in 4 (5 per cent) INT, 15 (13.8 per cent) PB and 3 (11.1 per cent) mixed type patients. R1 resection was observed in 7 (8.8 per cent) INT, 19 (17.4 per cent) PB and 6 (22.2 per cent) mixed subtype patients (*P* = 0.064).

In terms of postoperative complication rate, no statistically significant differences were observed. The 90-day mortality rate was significantly different: 2.5 per cent (*n* = 2), 10.1 per cent (*n* = 11) and zero in the INT, PB and mixed subtype respectively (*P* = 0.035).

### Overall survival

**Fig. 1:**
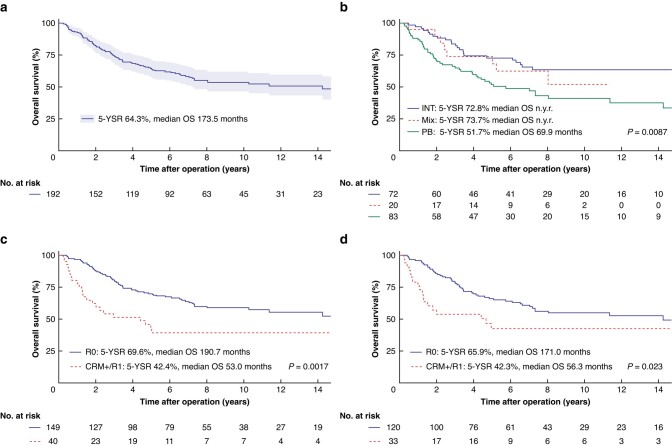
Postresection survival of ampullary cancer patients and effect of surgical radicality Overall survival rate of all resected ampullary cancer (AC) patients (*n* = 192) **a** Overall survival rate comparing the three histological subcategories INT (blue line), Mix (red line) and PB (green line); **b***n* = 175. Overall survival rate of all resected AC patients (*n* = 189); **c** and those with an INT or a PB subtype (*n* = 153); **d** with clear resection margins (R0 CRM–, blue line), or positive resection margins (R0 CRM+ or R1, red line); patients with missing information on R status were excluded. Patients alive at the point of last follow-up were censored. Patients with available follow-up of less than 90 days and those with distant metastases were excluded. *P* <0.05 was determined as the level of significance. OS, overall survival; INT, intestinal; PB, pancreatobiliary; Mix, mixed type; CRM, circumferential resection margin; 5-YSR, 5-year survival rate; n.y.r., not yet reached

Median follow-up was 50 months (i.q.r. 11–98). Five-year OS was 64.3 per cent (*n* = 192) with a median survival of 173.5 months (*Fig. 1a***)**. Analysis of the histological subtypes (*Fig. 1b*) showed a significant difference in 5-year OS in the three subgroups.

Analysis of all patients showed a significant difference in OS between true R0 resection and CRM+/R1 resection (*Fig. 1c*). The difference in OS remained when INT and PB subtypes were analysed only (*Fig. 1d*).

### Univariable and multivariable analysis

Some 42 patients were excluded from survival analysis due to the presence of distant metastasis or a follow-up shorter than 90 days. In the remaining 192 patients higher age, T status (pT4) and pN+ were significantly associated with worse survival in univariable analysis (*Table 3*). Adjuvant therapy was not significantly associated with postresection survival in univariable analysis. Missing information in a considerable number of patients precluded further analysis of this factor. Neoadjuvant therapy was significantly associated with postresection survival; the low number of patients precluded further analysis. A positive LN (PLN) ratio was significantly associated with worse survival. True R0 resection was directly associated with survival (HR 2.13; c.i. 1.31–3.46, *P* = 0.002). R0 CRM+ was significantly associated with worse survival (HR 3.24; c.i. 1.65–6.38, *P* <0.001).

**Table 3:** Univariable Cox regression analysis of parameters associated with overall survival (*n* = 192)

Parameter	Category	HR	95% c.i.	*P*
Age at operation (years)		1.03	1.00–1.05	0.018*
R status	R0 CRM–/R0 CRM+	3.24	1.65–6.38	< 0.001*
	R0 CRM−/R1	1.70	0.93–3.10	0.087
	R0 CRM−/(R0 CRM+ plus R1)	2.13	1.31–3.46	0.002*
Tumour size (cm)		0.93	0.77–1.11	0.392
ELN, *n*		0.99	0.97–1.02	0.513
PLN (of ELN), %		1.04	1.02–1.05	<0.001*
Whipple procedure	no/yes	1.26	0.65–2.44	0.495
Histological subtype	INT/mixed type	1.35	0.60–3.03	0.463
	INT/n.a.	0.92	0.38–2.27	0.863
	INT/PB	2.11	1.28–3.47	0.004*
T stage	T1/T2	0.93	0.40–2.16	0.866
	T1/T3	1.80	0.75– 4.35	0.190
	T1/T4	2.70	1.19–6.11	0.017*
	T1/Tis	0.55	0.07–4.48	0.576
N stage	pN0/pN+	3.45	2.15–5.54	<0.001*
POPF	yes/no	0.55	0.29–1.03	0.062

HR, hazard ratio; CRM, circumferential resection margin; ELN, number of examined lymph nodes; PLN, number of positive lymph nodes; POPF, postoperative pancreatic fistula; INT, intestinal; PB, pancreatobiliary; *statistically significant; n.a., not available/applicable.

In multivariable analysis (*Table 4*), both PLN ratio and pN+ were confirmed as independent variables significantly associated with worse postresection survival (*P* = 0.006 and *P* = 0.04 respectively).

**Table 4:** Multivariable Cox regression analysis of parameters associated with overall survival (*n* = 192)

Parameter	Category	HR	95% c.i.^†^	*P*
Age at operation (years)		1.03	1.00–1.05	0.052
PLN (of ELN), %		1.02	1.01–1.04	0.006*
R status	R0 CRM–/R0 CRM+	1.70	0.81–3.58	0.165
	R0 CRM−/R1	0.65	0.31–1.40	0.272
N stage	pN0/pN+	1.88	1.03–3.41	0.045*
Histological subtype	PB no/yes	1.39	0.87–2.21	0.173
T stage	T4 no/yes	1.74	1.00–3.11	0.063

HR, hazard ratio; CRM, circumferential resection margin; ELN, number of examined lymph nodes; PLN, number of positive lymph nodes; PB, pancreatobiliary; *statistically significant; ^†^calculated by approximation.

## Discussion

This study focused on the perioperative and long-term outcome of patients undergoing surgery for AC and the impact of radical resection on postresection survival.

Histological subtype is a known risk factor for postresection survival of AC. In a retrospective analysis of 170 resected patients, Zimmermann *et al*. showed a significantly worse 5-year OS rate of 27.5 per cent for PB, compared with 61 per cent for the INT subtype. The 5-year OS rate for mixed subtype was 44.4 per cent^6^. In this study, a generally better OS was seen for all subtypes, but differences were similar. The PB subtype was found to be an independent factor for worse postresection survival in multivariable analysis by several authors^5–7,26^. Kim *et al*. also found in their retrospective analysis of 104 resected AC patients, that tumours with the PB subtype presented with significantly more advanced T stage, and more LN metastases and perineural invasion^5^, which is in line with the present findings.

In recent years, research has focused on pN+ and LNR as risk factors for postresection survival. Some studies have identified pN+ status as an independent risk factor^6,27^, while LNR was not associated with worse survival in multivariable analysis^28,29^, in contrast to other findings^11,27,30^. Furthermore, some studies confirm the number of positive LN rather than the nodal status as a significant independent factor associated with worse survival^10,30^. In the present study, nodal status was confirmed as an independent risk factor for worse survival in multivariable analysis.

Data focusing on the R status with regards to survival of resected AC patients is limited. In an analysis of Sakata *et al*. on 71 patients undergoing PD, positive resection margin was significantly associated with worse survival in univariable analysis. In this analysis R1 and R2 were evaluated as one category^29^. Zimmermann *et al*. found R1 status to be independently associated with worse OS in 170 AC patients^6^, but there were only 8 patients with an R1 status available for analysis. No analysis was performed with regards to the CRM status. In the current analysis, no patient had gross macroscopic residual disease (R2); further R0 CRM+ was evaluated as a separate category and found to be significantly associated with worse OS in Kaplan–Meier analysis and univariable analysis compared with R0 CRM–. True R0 resection compared with CRM+/R1 status was associated with significantly better OS, but it was not confirmed in the multivariable analysis. Most likely, the numbers of R0 CRM+ (*n* = 23) and R1 (*n* = 33) were too low to reach statistical differences.

The main limitation of this study was its retrospective nature. As a surgical series, data might be biased by referral rate and selection of patients for surgery. Data on adjuvant therapy were available in less than 70 per cent of patients. No robust conclusion can be drawn about its clinical impact on survival.

This analysis underlines that the PB subtype is associated with more aggressive features such as higher tumour stage, poorer differentiation, higher rate of pN+ and worse prognosis compared with the INT subtype. A significant difference in OS of resected AC patients according to the CRM status was identified. Detailed histopathological workup, including the CRM status, should also become common practice for AC, as it already is for PDAC. Radical resection, defined as a ‘true’ R0 (CRM–) resection, is a key prerequisite for excellent 5-year OS in AC patients and should be performed regardless of the histological subtype.

## Data Availability

The data sets generated during and/or analysed during the present study are not publicly available, but are available from the corresponding author on reasonable request.
